# Research on Village Type Identification and Development Strategy under the Background of Rural Revitalization: A Case of Gaochun District in Nanjing, China

**DOI:** 10.3390/ijerph19116854

**Published:** 2022-06-03

**Authors:** Lingling Dai, Weifeng Qiao, Ting Feng, Yuanfang Li

**Affiliations:** 1 School of Geography, Nanjing Normal University, Nanjing 210023, China; 211302196@njnu.edu.cn (L.D.); ravenff@163.com (T.F.); 211302189@njnu.edu.cn (Y.L.); 2 Jiangsu Center for Collaborative Innovation in Geographical Information Resource Development and Application, Nanjing 210023, China

**Keywords:** village type, development level, reconstruction intensity, development strategy, Gaochun District in Nanjing, China

## Abstract

In the context of rural revitalization, it is of great significance for the implementation of a Rural Revitalization Strategy to carry out the research on scientifically identifying village types and clarifying the differences and pluralistic trends within villages. Taking Gaochun District of Nanjing in China as an example, this paper constructs an index system of development level and reconstruction intensity from a dynamic and static perspective, uses the polygon area method to calculate the comprehensive score of each index, divides village types based on the combination of development level and reconstruction intensity, and then puts forward the differentiated development strategies of various villages. The results show that the identification method of village types based on combined features is multi-dimensional and comprehensive, and the recognition results are more in line with the objective reality. Villages in Gaochun district have a medium overall development level and high overall reconstruction intensity. There are a large number of low-value villages with development level and high-value villages with reconstruction intensity. According to the three-step strategy of village type identification, the list of characteristic villages, the location of villages and the characteristics of index combination, five village types were identified: the characteristic protection type, the urban-suburban integration type, the agglomeration and upgrading type, the improvement and development type, and the relocation and merger type.

## 1. Introduction

With expansion and development centered on cities, many developed countries are facing the problem of rural decline, which had already appeared in the Europe’s Industrial Revolution in the eighteenth century [1]. Therefore, foreign countries have proposed solutions for rural development [2]. In the context of rapid urbanization, the contradiction of land use in rural areas has become increasingly prominent. In the process of transition, there are a series of phenomena of land use and rural function imbalance, such as farmland conversion and environmental pollution. The countryside is still a relatively weak link in China’s modernization drive [3]. In recent years, China has recognized the need to coordinate urban and rural development in the process of urbanization. Therefore, China has put forward the concept of a Rural Revitalization Strategy [4] to solve the problem of rural development and realize the integrated development of urban and rural areas. The Rural Revitalization Strategy includes economic, political, cultural, ecological and well-being construction. Its core purpose is to systematically construct the coupling pattern of population, land, industry and other development elements [5]. In essence, it is a systematic process of rural regional system element reorganization, spatial reconstruction and function improvement [6] that is designed to realize the comprehensive revitalization of the countryside. Villages carry the agglomeration of rural elements and spatial organization. A good village and town construction pattern is an indispensable spatial carrier for the integrated development of urban and rural areas [7]. Carrying out village classification research is the premise and foundation of optimizing the layout of villages and towns. In this context, how to identify village types to effectively support the implementation of a Rural Revitalization Strategy, realize the integrated development of urban and rural areas, and solve the problem of rural decline is the focus of this paper.

The research of village type identification is an important means of comprehensive dimensional cognition of the whole village. It is mainly classified by analyzing the internal and external multi-dimensional characteristics of the village and grasping the key differences. At present, village classification has become one of the hot issues in rural geography. Scholars at home and abroad have carried out a lot of research on village classification. As early as the 19th century, Albert Demangeon elaborated on different types of villages in France [8]. As the benchmark of rural construction, Britain is one of the countries that studied rural issues earlier in the world [9]. Since the 1970s, scholars have tried to understand the essence of rural economic differences and transformation, as well as the trend of social and economic diversification [10]. In terms of methods, it is optimized from the early single linear summation [11] to the combination of qualitative and quantitative [12]. GIS technology is then used [13,14]. The quantitative identification of village type division mainly focuses on the perspective of rural evaluation [15,16,17], and the spatial layout optimization and the evolution characteristics of different agricultural settlements from the perspective of function [18,19]. By analyzing the changes of spatial structure in rural areas [20], it formulates rural development plans and puts forward rural structure optimization strategies [21,22,23].

Domestic research on village classification mostly starts from the perspective of sociology and geography. From the perspective of sociology, most are classified studies under the background of differences in rural social structure [24]. Under the multi-perspective of rural geography, most are the recognition of regional differences. Previous studies on village classification mostly focused on a certain dimension of village development and used single factors to divide village types, such as economic level [25,26,27,28,29], geographical characteristics [30,31], and location characteristics [32,33]. However, village development is a multi-dimensional process, which cannot be measured by a single index. There is a complex relationship among the natural, economic and social factors affecting the development and construction of villages. Since the Rural Revitalization Strategy was put forward, scholars have carried out multi-dimensional comprehensive identification of rural types from the micro scale. Qiao Luyin [34] built a rural type identification system with four dimensions including natural factors, location conditions, homestead utilization and resource endowment. Based on in-depth research on the theory of urban-rural integration development, Wen Qi [35] constructed four types of identification subsystems, namely rural entity, industrial development, living environment, and resource endowment. Li Yurui [36] established a village classification model (VCM) to clarify the division method of four types of villages in rural revitalization according to the needs of practical work. By adopting the comprehensive evaluation method of development and reconstruction, Han Xinyu [37] constructed the index system of village development degree and reconstruction degree, and divided the types of villages through their high and low clustering. In terms of research methods, many scholars use the comprehensive index evaluation method [38,39,40] and systematic clustering method [37] to divide village types. Some scholars use a spatial superposition analysis method [41], a gravity center migration model [42] and a logistic geographic weighted regression model [13] to reveal the spatial differentiation characteristics and spatial evolution laws of village types.

The previous studies on village classification were mostly based on a static perspective, but the development of villages has continuity. The classification of villages not only needs to consider the differences of various current factors that affect the development of villages, but also the development process of villages. The development process itself reflects the advantages and disadvantages of village development and has great reference value. In view of this, based on the relevant national planning requirements, this paper makes full use of existing classification schemes, selects the Gaochun District in Nanjing as an example, and explores the ideas and methods of village type identification from the perspective of dynamic and static combination in order to provide theoretical support and decision-making reference for scientifically carrying out village classification and compiling village planning so as to help rural revitalization.

## 2. Materials and Methods

### 2.1. Study Area and Data Collection

**Figure 1:**
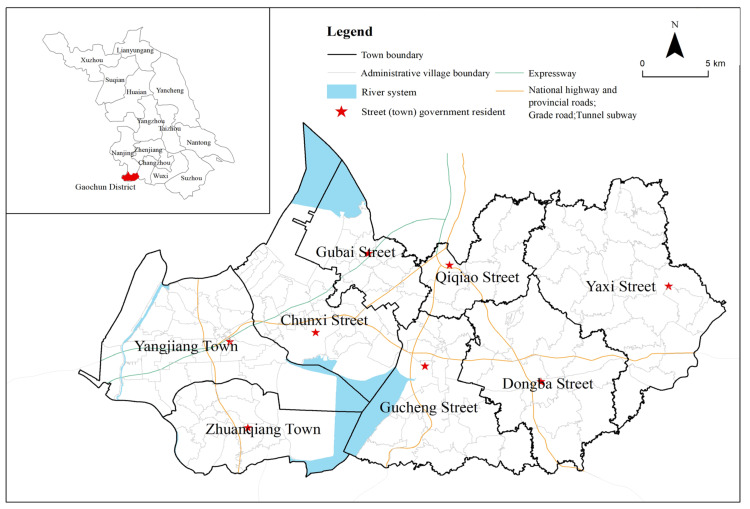
Research area.

Gaochun District is located at the southwestern end of Jiangsu Province, the southern end of Nanjing City, at the junction of Jiangsu and Anhui provinces. The whole district is 790.23 km^2^, and the registered residence population was 449,300 at the end of 2019. The government is responsible for six streets and two towns, with a total of 129 village (resident) committees. The terrain is high in the east and low in the west. The east is the junction of Maoshan Mountain and Tianmu Mountain. Most of the rivers in the low mountains and hills in the east flow eastward into Taihu Lake. The river network is not dense and sparse. The West belongs to the lacustrine plain of Gucheng Lake, Shijiu lake and Danyang Lake. The river flows westward into the Yangtze River, and the river network is dense. Gaochun District is located within the one-hour metropolitan area of Nanjing. Liwu Expressway and Wutai Highway run through Gaochun from east to west, Ninggao Expressway and Gaoxuan Expressway run through north and south, and Ninggao New Channel and Ninggao Intercity Rail Transit directly connect Nanjing across Shijiu Lake. Gaochun District is a characteristic urban agricultural production area in East China, with regional characteristic industries such as crab and edible fungi. At present, it is preliminarily developed into a high-efficiency agricultural agglomeration area. Gaochun District is rich in natural landscapes and historical and cultural resources. In 2010, Yaxi was officially awarded the title of “International Slow City”, becoming the first international slow city in China. The architectural style of Gaochun Old Street in the Ming and Qing Dynasties is completely preserved, and it enjoys the reputation of “the first ancient street in Jinling”. The current development of rural leisure tourism and homestays is one of the distinctive features of Gaochun District’s tourism resources (Figure 1).

The data of rural development and evolution in this paper are from the survey data of land use change in Gaochun District in 2009 and 2018. The current administrative village division of Gaochun District is from the third national land survey. The socio-economic data of the village comes from the statement of the basic social and economic situation of the village filled in by each administrative village and formulated by the National Bureau of Statistics. Due to the different filing details of each village, it is difficult to collect village statistical data. This paper selects the data of 2015 and 2018, and the relevant data are missing in three villages. In the data preprocessing of the development level indicator system, the road network data used in the accessibility analysis is derived from the monitoring data of the national census of basic geography of Gaochun District in 2019. The land use change survey data in 2015 and 2018 were uniformly used for the change of per capita arable land area and the change of forest coverage. In the preprocessing of the village reconstruction intensity index system, the measurement data of the planned ecological location comes from the 2020 version of the ecological protection red line of the Gaochun District.

### 2.2. Research Framework

Under the influence of urbanization and industrialization, China’s urban and rural society has undergone a dramatic transformation, and the relationship between man and land has changed significantly [43]. As the outflow area of population, capital and other factors, the social, economic and spatial organization of rural areas have been reconstructed to varying degrees. Rural development is a unified process of long-term operation and the phased reconstruction of the regional social economy. The long-term development is the premise and motivation of reconstruction, and the reconstruction activities in a certain period of time are the real-time feedback to the development [37]. The level of village development refers to the development state of the rural regional system measured at a specific time node. Village reconstruction refers to the changes of rural regional form and socio-economic level under the influence of social, economic, natural and other factors, which promotes the continuous change and reorganization of the rural regional system structure. The intensity of village reconstruction changes with different time periods.

Referring to the existing research [37,44], this paper intends to classify villages from static and dynamic perspectives, and puts forward the construction of two index systems of village development level and reconstruction intensity to grasp the overall characteristics of rural areas. Among them, the measurement of village development level mainly finds out the actual state of the village and pays attention to the development conditions. The measurement of village reconstruction intensity focuses on the change intensity of villages in the past and the revitalization potential in the future.

Following the law of rural development, this paper refers to the village classification framework at the national and provincial levels, known as the “Strategic Planning for Rural Revitalization (2018–2022)”, in addition to the village classification ideas of some scholars [36]. Given the difficulty of identifying village types, this paper identifies the types of villages in three steps, and divides the village types into five types, including the characteristic protection type, the urban-suburban integration type, the agglomeration and upgrading type, the improvement and development type, and the relocation and merger type. Among them, the urban-suburban integration villages refer to the villages located in the suburbs of the city and the location of the county towns, and has the conditions to transform into towns. The agglomeration and upgrading villages are the existing large-scale central villages, which have a certain radiation and driving effect, and a high development level and reconstruction intensity. The improvement and development villages are villages with a certain scale, but there are certain development shortcomings in the process of rural development.

**Figure 2:**
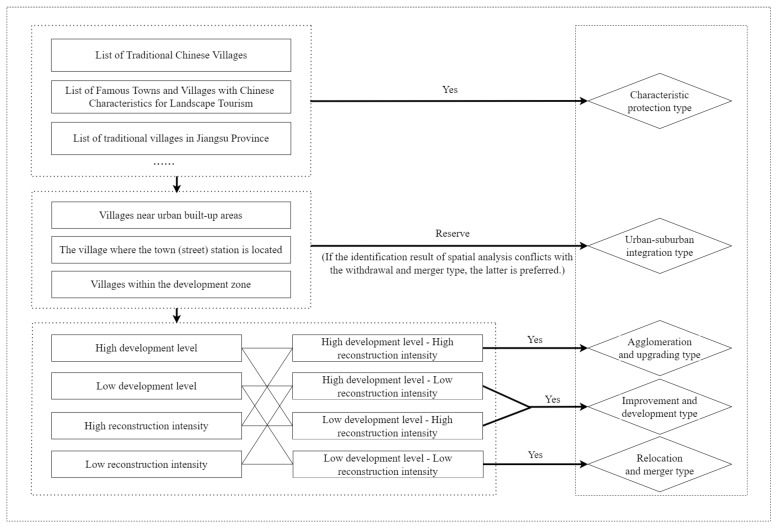
Framework of village type identification.

The specific identification steps are as follows. Firstly, characteristic villages are an important carrier of historical and cultural inheritance, and have priority for key protection. According to the “List of Traditional Chinese Villages”, the “List of Famous Towns and Villages with Chinese Characteristics for Landscape Tourism” and other information, they determine whether the villages belong to the characteristic protection type. Secondly, according to whether the village is located near the urban built-up area, the area where the town (street) is located, or the development park, it is judged whether the village belongs to the urban-suburban integration type. Finally, through the combination characteristics of development level and reconstruction intensity, the identification is carried out in the order of the agglomeration and upgrading type, the improvement and development type, and the relocation and merger type. Specifically, the combined characteristics of high development level and high reconstruction intensity belong to the agglomeration and upgrading type. The combined characteristics of high development level, low reconstruction intensity and low development level, high reconstruction intensity is classified as the improvement and development type. And the combined characteristics with low development level and low reconstruction intensity is judged as the relocation and merger type. It should be noted that if the identification of the urban-suburban integration type identified by spatial analysis conflicts with the identification of the relocation and merger type, the village will be identified as the relocation and merger type first. Because the village lacks development prospects at this time, it should be withdrawn and merged (Figure 2).

### 2.3. Methods

#### 2.3.1. Construction of Index System

(1) Initial structure of index system

Combined with the theory of the rural regional system and the theory of urban-rural integrated development, it can be seen that there are many factors affecting the level of rural development which are nature, economy, society and other aspects. On the basis of referring to the “Strategic Planning for Rural Revitalization (2018–2022)” and related research [34,35,36,37], combined with the characteristics of rural development in Gaochun District, this paper constructs the village development level index system (Table 1). It covers the village scale, location condition, industrial development, natural resources and the public service of five dimensions, focusing on the static description of the long-term development accumulation and current situation of the village.

Fundamentally speaking, rural reconstruction is a positive development process of the rural regional system which is manifested as a transition from a non-benign state to a benign state. In other words, villages realize the process of improving the quality of rural functions through their cumulative quantitative changes [45]. Based on the significant rural spatial development characteristics of the study area, this paper constructs the village reconstruction intensity index system (Table 2) covering three dimensions, including village scale, industrial development and natural resources. The evaluation of reconstruction intensity focuses on revealing the characteristics of changes in the structure of internal and external elements in the process of rural transformation. By comparing the village status at the beginning and the end of the period, we can obtain indicators of the reconstruction and positive improvement of the village during the study period. Strictly speaking, rural reconstruction is a relatively dynamic concept, and changes in time nodes will cause corresponding changes in reconstruction intensity [46].

(2) Simplification and finalization of the index system

Due to the large number of indicators of village development level and the existence of collinear indicators, the factor analysis method of SPSS software was used to simplify the indicators, including preliminary testing, correlation analysis, factor extraction and factor naming. Eight criteria levels and fifteen indicators are obtained. The Criterion level includes location construction conditions, economic construction level, village construction scale, natural environment endowment, integration characteristics of three-industries, living standards, the scale agriculture level and the social security level (Table 3). When the village reconstruction intensity index was tested by the KMO and Bartlett’s spherical test, it was found that it did not meet the conditions for factor analysis. This shows that there is no obvious collinearity between the constructed indicators, so the simplified analysis of indicators was not done.

#### 2.3.2. Index Weight Calculation

Due to the strong subjectivity of AHP, this paper adopts the combination of AHP and entropy weight method to determine the final weight. The subjective weight is first determined by the AHP method. The objective weight calculated by the entropy weight method is used to correct the subjective weight, and then the concept of distance function is introduced. The combined weight of the indicators is calculated by the linear combination method to reduce the interference caused by the large fluctuation data so that the difference between subjective weight and objective weight is consistent with the difference between distribution coefficients (Table 2 and Table 3).

#### 2.3.3. Calculation Based on Polygon Area Method

**Figure 3:**
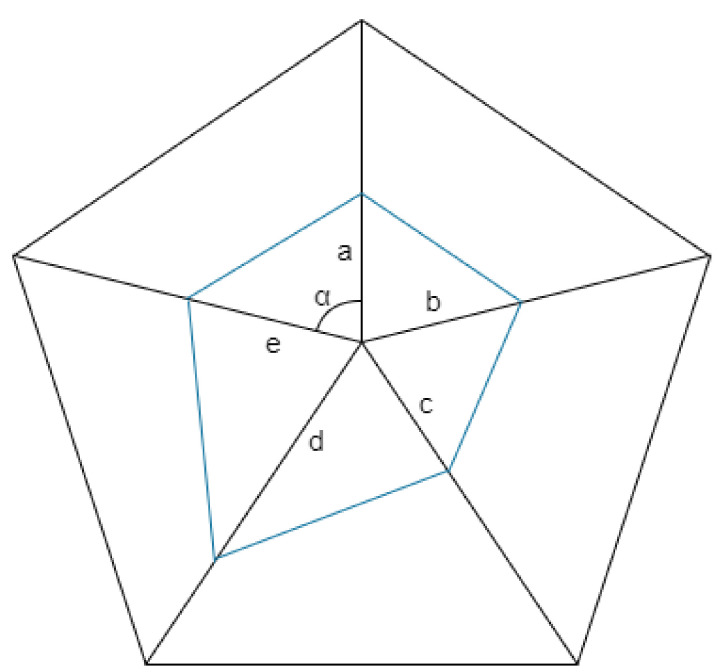
Schematic diagram of polygon area method.

The healthy development of a village is the combination of comprehensiveness and sustainability. The high score for a certain development dimension and a high total score obtained by weighting do not fully explain the overall good comprehensive development of the village. Therefore, considering that rural development is a comprehensive process affected by multiple factors, this paper abandons the simple weighting method used in calculating index scores in the past, and chooses to use the method of calculating polygon area [34] to determine the comprehensive scores of village development level and village reconstruction intensity respectively (Figure 3). The specific calculation method is:

(1) $$S=\left(ab+ba+cd+de+ea\right)\times sin\frac{\alpha }{2}$$

In Formula (1), *a*, *b*, *c*, *d*, *e* is the single Criterion level score, and α is the angle between any two indicators.

Since the area calculation results of different sorting methods are different, we choose to average the area of all polygons composed of multiple indicators. It can be obtained from the calculation that the average size of the combined area of all possible polygons in multiple dimensions depends on the added value of the multiplication of multiple index scores, which is defined in this paper as the index polygon value P.

(2) P=ab+ac+ad+ae+bc+bd+be+cd+ce+de

## 3. Results

### 3.1. Result Analysis

**Figure 4:**
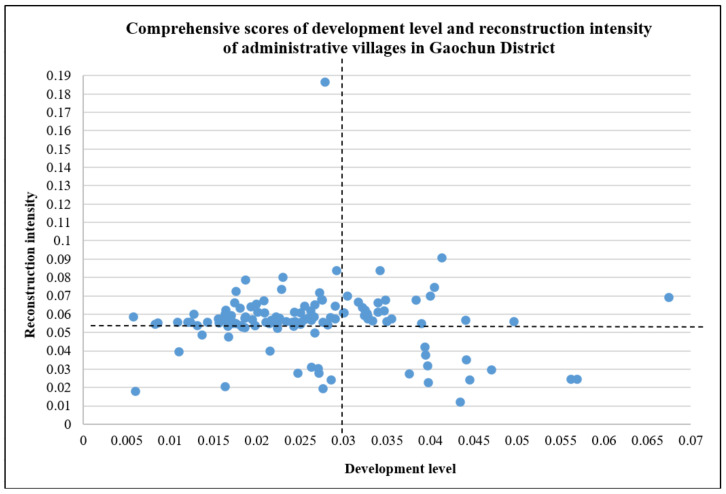
Scatter plot of comprehensive scores of development level and reconstruction intensity of administrative villages in Gaochun District.

The comprehensive score of development level reconstruction intensity of 126 administrative villages in the Gaochun District is calculated by the polygon area method, and it is made into a scatter diagram (Figure 4).

#### 3.1.1. Analysis of Village Development Level in Gaochun District

**Figure 5:**
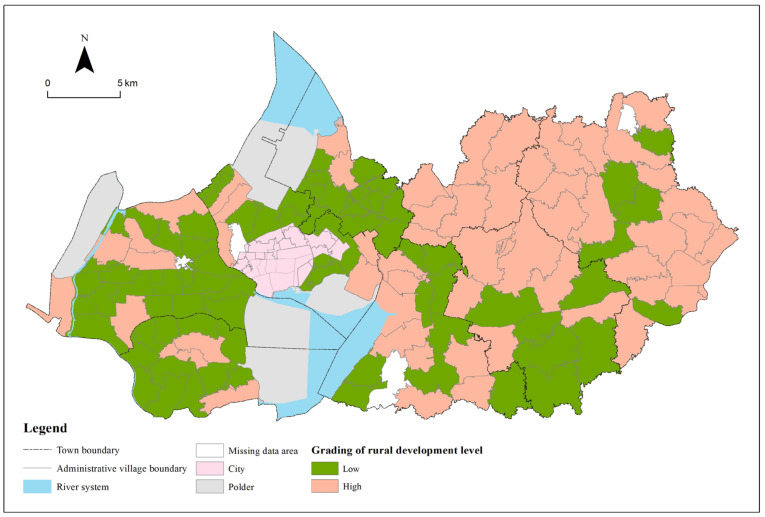
Development level distribution of administrative villages in Gaochun District.

In ArcGIS 10.2, the development level of 126 villages is divided into two categories by the natural discontinuity method, of which 0–0.027 is a low score, and 0.027–0.07 is a high score (Figure 4). The overall development level of administrative villages in the Gaochun District is not high. Most of the villages have a low development level score. There are 73 low-value villages and 53 high-value villages, with an average score of 0.026. The development level of 126 villages in the Gaochun District is visually expressed on the map (Figure 5). The high development level is mainly distributed in Qiqiao Street, Yaxi Street, Dongba Street and Gucheng Street. The overall development of Qiqiao Street is relatively good. There are no low-level development villages within the street. Most of Yaxi Street consists of high-value development villages. The villages in the north of Dongba Street, which account for about half of the street, belong to the high-level development area. Except for the villages in the middle of Gucheng Street, the remaining villages belong to the high-value development areas, accounting for about half of the number of administrative villages under the jurisdiction of Gucheng Street. The low-value areas of development level are mainly distributed in Yangjiang Town, Zhuanqiang Town, Chunxi Street and Gubai Street. The measurement of village development level includes multiple dimensions, but Yangjiang Town and Zhuangqiang Town in the east are dominated by agricultural production (aquaculture), and the driving force for development is relatively single, resulting in a low level of development. Most of Chunxi Street and Gubai Street belong to urban built-up areas. Although they occupy excellent location advantages, the regional development of villages will be restricted by urban development to a certain extent. For example, the overall development level is not high due to the limited area of villages.

#### 3.1.2. Analysis of Reconstruction Intensity of Villages in Gaochun District

**Figure 6:**
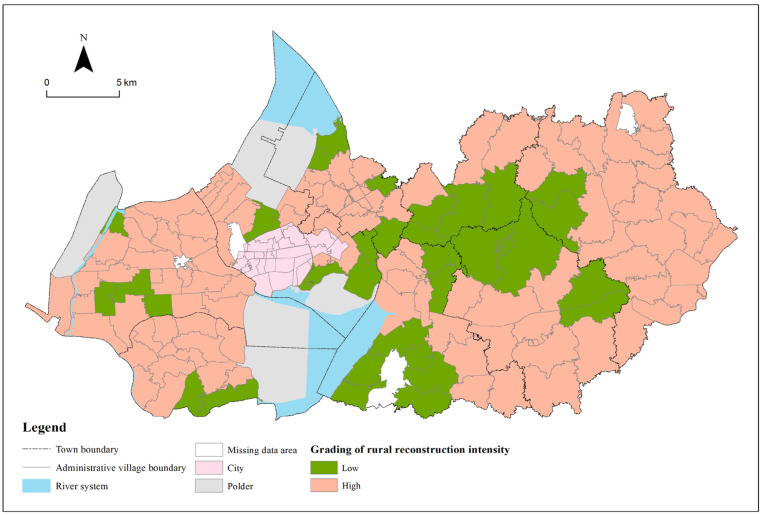
Distribution of reconstruction intensity of administrative villages in Gaochun District.

In ArcGIS 10.2, the reconstruction intensity of 126 villages was divided into two categories by the natural discontinuity method, of which 0–0.054 belonged to a lower score, and 0.054–0.190 belonged to a higher score. The reconstruction intensity scores of administrative villages in Gaochun District are generally high, with 33 low-value villages and 93 high-value villages, with an average score of 0.056 (Figure 4). The reconstruction intensity of 126 villages in Gaochun District was visualized on a map (Figure 6). The high-value areas of reconstruction intensity were mainly distributed in Yangjiang Town, Zhuanqiang Town, Yaxi Street, Dongba Street, and Gubai Street. It shows that these towns (streets) have a good positive development trend in recent years, and the villages have great potential for development. In the future, if they are correctly guided, they will continue to improve in a positive direction. The low-value areas of reconstruction intensity are mainly distributed in the south of Qiqiao Street, the north of Dongba Street and the south of Gucheng Street. These three places have better natural endowments and pay attention to ecological protection. Especially under the background of promoting ecological civilization construction in recent years, the development on the premise of sacrificing the environment is not significant in Gaochun rural areas, but the rural social and economic development potential is poor. Focusing on this characteristic can help guide the development into a certain characteristic village, such as an industrial characteristic village, a cultural characteristic village, and so on.

### 3.2. Classification of Village Types

**Figure 7:**
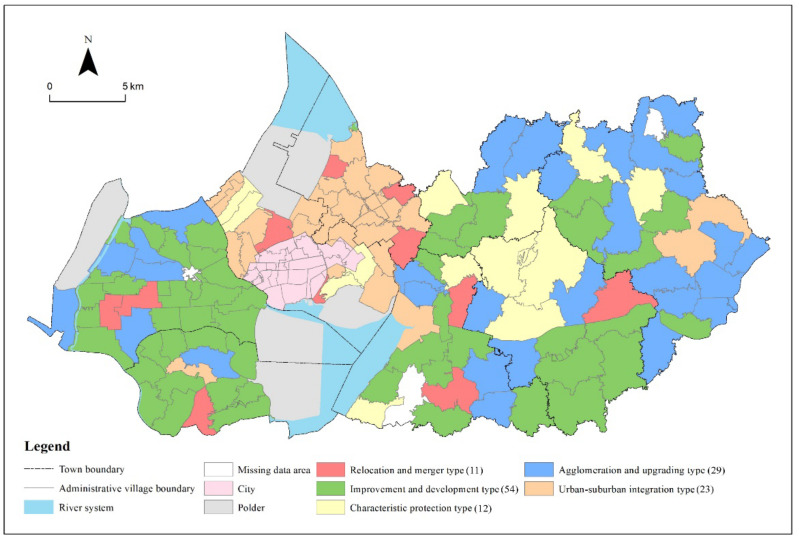
Results of village type identification in Gaochun District.

According to the classification results of the two indicators of development level and reconstruction intensity, it can be divided into four areas in the comprehensive scatter plot of development level and reconstruction intensity of administrative villages in Gaochun District. Based on the framework of village type identification in Figure 2, the administrative villages in the four areas are divided into low development level-low reconstruction intensity, low development level-high reconstruction intensity, high development level-low reconstruction intensity, and high development level-high reconstruction intensity. Combining the first and second steps of the village type identification framework, five village types are identified (Figure 7).

(1) Characteristic protection villages

Such villages are rich in cultural connotations, mainly referring to whether they are included in historical and cultural villages, traditional villages, ethnic minority villages, famous scenic tourism villages, characteristic countryside and so on, to clarify whether they are classified as characteristic protected villages. In order to effectively inherit the history and culture, such villages should be protected as much as possible. Considering the protection of rural cultural ecology as a whole, the key is to refine the classification of characteristic protection. By compiling cultural industry planning in line with rural characteristics, villages with characteristic natural and human resources and unique local products should cultivate dominant industries and develop the tourism industry appropriately.

(2) Urban-suburban integration villages

Such villages are mainly composed of villages near urban built-up areas, villages where the town (street) government is located and villages within the development zone, excluding villages that have been identified as characteristic protection villages and later identified as relocation and merger villages. Such villages have close communication with towns and development zones and superior location conditions. They have the advantage of becoming the back garden of towns and the conditions to transform into towns. In the future, we can fully develop the unique resource advantages of the region and determine its development direction can be determined according to the size and level of the town. The infrastructure and public service facilities of towns should be improved, and the connection and functional coordination with cities and streets should be done well.

(3) Agglomeration and upgrading villages

In this paper, villages that satisfy both high development level and high reconstruction intensity are divided into the agglomeration and upgrading villages. From the perspective of spatial distribution, it is mainly distributed in areas with a relatively developed traffic network, and the overall development of rural areas is better. From the town level, it is mainly distributed in Yaxi Street and Yangjiang Town. Such villages have a relatively good level of economic development, a good resource base and significant location advantages, which are suitable for guiding positive improvement. Guaranteeing rural public facilities and services is one of the key points. Therefore, it is necessary to use its own resource base to balance the allocation of public resources between urban and rural areas, give play to the urban radiation effect and the agglomeration effect of villages, and at the same time undertake the population that has moved out of the surrounding villages and the reverse floating population of some cities to build a new type of village as a spatial carrier for the survival, consumption and work of the migrant population.

(4) Improvement and development villages

Such villages consist of villages with high development level and low reconstruction intensity, and low development level and high reconstruction intensity. That is, although the village has a certain radiation driving role, in the process of rural development, the lack of stock resources such as population, capital and land leads to the limited development power, there are certain shortcomings, and the development needs of structural function adjustment and optimization are faced. From the perspective of spatial distribution, such villages are widely distributed and account for the highest proportion in Gaochun District, accounting for about 40% of the number of administrative villages. From the town level, in addition to Chunxi Street and Gubai Street, other towns (streets) are involved, with more distribution in Yangjiang Town and Brick Wall Town. Gaochun District, as an outer suburb agricultural county for many years, has the problem of aging and weakening the main body of villages in the process of development. And such villages lack distinctive characteristics in development, so the key is to create industrial clusters with distinctive regional characteristics, vigorously develop a collective economy based on agricultural foundations and adapt to market demands, and increase the attractiveness of villages through the integrated development of production and villages. By bringing talents back to their hometown, they will activate the endogenous driving force of villagers to assist in development and reduce the loss of the village population and resources.

(5) Relocation and merger villages

In this paper, the villages with low development level and reconstruction intensity are divided into the relocation and merger villages. From the perspective of spatial distribution, such villages are interspersed between the agglomeration and upgrading villages and the improvement and development villages. From the perspective of the town scope, the relocation and merger villages are scattered in Yangjiang Town, Chunxi Street and Gubai Street. Such villages have long been limited in size and slow in industrial transformation, lack development power, have weak economic growth, and significantly lag behind the overall regional level in social and economic development, resulting in low land use efficiency, the obvious hollowing out of villages, failure to improve the natural environment, serious population loss, a high proportion of migrant workers, a lack of residents’ sense of well-being, etc.. For the relocation and merger villages, there are various reasons for merger and withdrawal. It is necessary to deeply analyze the reasons for the merger and withdrawal, and to insist on classification to promote the future construction and development of the countryside. First, for villages that were withdrawn due to poor infrastructure and inconvenient living conditions, priority should be given to areas with flat terrain, few development restrictions and industrial development potential, and attention should be paid to making up for the shortcomings of the foundation. Secondly, for villages that need to be merged due to ecological and environmental protection, a combination of various relocation and reconstruction methods should be adopted, including the establishment of planning guidance and ecological compensation mechanisms. Thirdly, for villages that need to be relocated due to serious population loss and hollowing out, priority should be given to the paid withdrawal of the original villagers’ land and compensation for replacement.

## 4. Discussion

In the early stage of China, there were prominent problems such as unbalanced rural development and disorderly village construction, which led to the gradual widening of the gap between urban and rural areas. The Rural Revitalization Strategy is proposed to solve the increasingly serious problem of rural decline, and its importance is self-evident. At present, rural revitalization is progressing steadily, and the classification of village type has gradually become one of the hotspots of discussion and research. The division of village types is an indispensable link in the integrated development of urban and rural areas, and an important prerequisite for planning and policy formulation in the process of rural transformation and reconstruction. Only by scientifically identifying and dividing the types of villages can village development strategies be put forward in a targeted manner to solve the problem of unbalanced rural development, which has important practical significance. Under the background of Rural Revitalization, this paper takes the Gaochun District of Nanjing City, Jiangsu Province as an example. Jiangsu Province is a developed eastern coastal province. Gaochun District is located in the outer suburbs of Nanjing. In recent years, in the process of rapid urbanization and the transformation of land use has become prominent, and the differentiation of villages has become increasingly apparent, with obvious typicality. As a characteristic urban agricultural place of origin, its rural development characteristics have strong representativeness. In addition, Gaochun District is rich in historical and cultural resources and tourism resources, and has won the title of “International Slow City”, which has a certain distinction. Therefore, there is important application value in classifying villages in the Gaochun District and proposing a revitalization path accordingly. Based on the current conditions of Gaochun District, according to the village development level and reconstruction intensity, this paper scientifically classifies the villages and proposes differentiated development strategies according to local conditions so as to provide a reference for local development direction.

In terms of village type division, “Strategic Planning for Rural Revitalization (2018–2022)” clearly puts forward four village types: the agglomeration and upgrading type, the urban-suburban integration type, the characteristic protection type, and the relocation and merger type. Based on this, scholars have carried out micro-scale research on the village type recognition method system. Based on the requirements of the document, this study uses the above research methods to divide villages into five types. The spatial distribution of each type of village is basically consistent with the actual situation. Previous studies on the elements of village internal development are still relatively insufficient. Furthermore, some existing classifications are inconsistent with the requirements of “Strategic Planning for Rural Revitalization (2018–2022)”. Overly diversified classification results are not conducive to macro-control. Compared with previous studies, this paper starts from the characteristics of the development and evolution of rural settlements based on the two dimensions of development level and reconstruction intensity, and combines the dual perspectives of static and dynamic to study the classification of villages, emphasizing the analysis of the inherent differences in village types.

Although there are some improvements in the framework and methods of village classification, there are still shortcomings. First, from the perspective of the applicability of the method, in the analysis process, this study refers to the “Strategic Planning for Rural Revitalization (2018–2022)” and related literature, and selects representative index factors, which have a certain applicability. However, there are obvious differences in natural endowments, economic development, urban and rural construction among provinces in China. Therefore, the index system is not directly applicable to research in other regions. Some evaluation indicators need to be adjusted or increased or decreased according to the actual situation of the region. The weight calculation method of the index comprehensively considers subjective and objective factors, which can provide a certain reference, but it still needs to be further improved. Second, from the perspective of data limitations and updates, this study uses village-level data from 2015 and 2018. Since the data is filled in by each village, it is difficult to obtain village-scale data, and the data collection process is difficult. There is a lack and insufficient timeliness of data. Therefore, the availability and timeliness of data must be considered in future research, so as to facilitate further research on village planning in the future. Third, from the perspective of classified policies, Gaochun District has certain advantages in terms of economic foundation and location conditions. The government should grasp the characteristics of village types, balance the relationship between village development and characteristic protective villages, strengthen the construction of village infrastructure, and maintain the development environment required for the secondary and tertiary industries of the village. The development strategy of this paper is put forward based on the current stage of village development. In the future, with the continuous changes of internal and external factors affecting the development of villages, the corresponding development strategies will also be adjusted accordingly to better guide the classified development of villages.

## 5. Conclusions

Under the background of the implementation of the Rural Revitalization Strategy, starting from the characteristics of the development and evolution of rural settlements, this paper divides the Gaochun District into five types step by step from the perspective of combining the dynamic and static, and then proposes differentiated development strategies for each type of village. The main conclusions of this paper are as follows:

(1) This paper constructs the index systems of development level and reconstruction intensity, uses the polygon area method to calculate the comprehensive score of the index, and classifies the combination of the two types of indexes according to the score results. The development level index system covers eight dimensions including location construction conditions, economic construction level, village construction scale, natural environment endowment, integration characteristics of the three industries, living standard, scale agriculture level, and social security level. The reconstruction intensity includes three dimensions including village scale, industrial development, and natural resources. Among them, the villages with high development level and high reconstruction intensity are determined as the agglomeration and upgrading type. The villages with high development level-low reconstruction intensity and low development level-high reconstruction intensity are identified as the improvement and development type. The villages with low development level and low reconstruction intensity are determined to be the relocation and merger type.

(2) With the help of the village type identification method proposed in this paper, according to the three-step strategy of village type identification, five types of villages in the Gaochun District were identified, including 12 characteristic protection villages, 23 urban-suburban integration villages, 29 agglomeration and upgrading villages, 54 improvement and development villages, and 11 relocation and merger villages. The overall development of villages in Gaochun District is at a medium level, and its overall reconstruction intensity is high. The village type is mainly the improvement and development type. In terms of distribution law, characteristic protection villages are mainly distributed in Qiqiao Street and Dongba Street with characteristic humanities and natural resources. The urban-suburban integration villages are mainly distributed in the suburbs of cities or around the county towns with a significant location advantage. The agglomeration and upgrading villages are mainly distributed in Yaxi Street and Yangjiang Town in areas with certain transportation advantages and relatively complete infrastructure. A large number of the improvement and development villages are widely distributed in the towns of Gaochun District. The relocation and merger villages are interspersed between the agglomeration and upgrading villages and the improvement and development villages.

(3) Under the background of rural revitalization, based on the five types of villages, differentiated development strategies for five types of villages are proposed. The characteristic protection villages should be focused on protection, supplemented by appropriate development, and formulate cultural industries or rural tourism plans in line with rural characteristics, as well as cultivating advantageous leading industries based on characteristic natural and human resources. The future development direction of the urban-suburban integration villages should be determined according to the scale and level of the town, and the connection and functional cooperation with cities and streets should be well done. The agglomeration and upgrading villages should give full play to their comprehensive advantages and increase investment in village infrastructure construction so as to undertake and gather the floating population in the surrounding villages or cities and strengthen the role of villages in radiating and driving the surrounding areas. The improvement and development villages should focus on development characteristics and promote the development of local characteristic industries to make up for the shortcomings in rural development. The relocation and merger villages should be promoted by classification and deeply analyze different relocation reasons to promote the future construction and development of the village in a targeted manner.

## Figures and Tables

**Table 1:** The village development level index system.

Target Level	Criterion Level	Index Level	Computing Method	Indicator Attributes
Village scale	Population size	Registered residence population	Basic social and economic situation of village in Gaochun District	+
		Resident population		+
	Land use scale	Village land area	ArcGIS raster computing	+
		Homestead per capita	Village land area/resident population	−
	Economic scale	Total collective assets of the village (10,000 yuan)	Basic social and economic situation of village in Gaochun District	+
		Annual collective economic income (10,000 yuan)		+
Geographic conditions	Macro-location	Distance to district government station	ArcGIS raster computing	−
	Micro-location	Distance to town (street) government		−
Industrial Development	Agricultural production	Facility agriculture scale	Basic social and economic situation of village in Gaochun District	+
		Cultivated land area under large-scale operation		+
		Breeding pit and pond area		+
		Number of irrigation ponds and reservoirs		+
	Three-industry integration	Number of agricultural products processing enterprises		+
		Number of farmers selling agricultural products online		+
		Number of farmers with business license engaged in leisure agriculture and rural tourism		+
	Income level	Per capita disposable income of villagers		+
Natural resources	Resource endowment	Per capita arable land	Village cultivated area/registered residence total population	+
	Ecological environment	Forest coverage rate	Village forest land area/administrative village area	+
Public service	Social security	Proportion of rural special poverty-stricken people	Number of rural poor/total number of registered residences	+
	Public utilities	Public infrastructure level	Types of public infrastructure/survey facilities	+
		Proportion of running water users	Number of running water users/number of registered residence	+
		Proportion of sanitary toilet users	Number of toilet users/the number of registered households	+

Note: In indicator attributes, "+" means positive index. And "−" means negative index.

**Table 2:** Weight of village reconstruction intensity.

Target Level	Criterion Level	Index Level	Indicator Attributes	Computing Method	Weight
Village scale	Population size	Population migration rate	+	Resident population/registered residence population	0.106
		Proportion of people left behind	−	Left behind population/registered residence population	0.027
	Land use scale	Homestead changes per capita	−	Difference value of per capita homestead value between the two phases	0.042
	Economic scale	Changes in total village collective assets	+	Difference value between total collective assets of villages in two phases	0.106
Industrial Development	Agricultural production	Changes in the number of ponds and reservoirs for irrigation	+	Difference value between the number of ponds and reservoirs for irrigation in the two phases	0.087
	Three-industry integration	Number of new business entities	+	Basic social and economic conditions of the village in Gaochun District	0.287
Natural resources	Resource endowment	Changes in per capita arable land	+	Difference value of per capita cultivated land area between the two periods	0.096
	Ecological environment	Change in forest cover	+	Difference value of forest coverage between two periods	0.076
		Planned ecological location	−	Ecological red line buffer zone	0.173

Note: In indicator attributes, "+" means positive index. And "−" means negative index.

**Table 3:** Weight of development level classification indicators.

Target Level	Index Level	Weight		
		AHP	Entropy Weight Method	Combination Method
Village scale	Registered residence population	0.055	0.008	0.038
	Resident population	0.057	0.007	0.036
	Village land area	0.027	0.092	0.051
	Homestead per capita	0.075	0.016	0.053
	Total collective assets of the village (10,000 yuan)	0.106	0.104	0.105
	Annual collective economic income (10,000 yuan)	0.036	0.001	0.023
Geographic conditions	Distance to district government station	0.050	0.109	0.072
	Distance to town (street) government	0.073	0.025	0.055
Industrial Development	Facility agriculture scale	0.038	0.075	0.052
	Cultivated land area under large-scale operation	0.058	0.150	0.093
	Breeding pit and pond area	0.034	0.237	0.111
	Number of irrigation ponds and reservoirs	0.185	0.007	0.118
	Number of agricultural products processing enterprises	0.116	0.018	0.079
	Number of farmers selling agricultural products online	0.063	0.080	0.070
	Number of farmers with business license engaged in leisure agriculture and rural tourism	0.027	0.071	0.044
	Per capita disposable income of villagers	0.055	0.008	0.038
Natural resources	Per capita arable land	0.057	0.007	0.036
	Forest coverage rate	0.027	0.092	0.051
Public service	Proportion of rural special poverty-stricken people	0.075	0.016	0.053
	Public infrastructure level	0.106	0.104	0.105
	Proportion of running water users	0.036	0.001	0.023
	Proportion of sanitary toilet users	0.050	0.109	0.072

## Data Availability

No new data were created or analyzed in this study. Data sharing is not applicable to this article.
